# Elimination of visceral leishmaniasis as a public health problem in Bangladesh: Lessons learned and questions remaining

**DOI:** 10.1371/journal.pntd.0013949

**Published:** 2026-02-24

**Authors:** Rajib Chowdhury, Anupama Hazarika, Sabera Sultana, Md Sakhawat Hossain, Shampa Saha, Shah Golam Nabi, Abu Nayeem Mohammad Sohel, Md Jewel Rana, Mizanur Rahman, Manzurul Haque Khan, Narayan Prosad Maheswary, Axel Kroeger, Aya Yajima, Dinesh Mondal, Sheikh Daud Adnan, Sanya Tahmina Jhora, Md Nazmul Islam, Ariful Basher, Abul Khair Mohammad Shamsuzzaman, Be-Nazir Ahmed, Md Abul Faiz, Jorge Alvar, Caryn Bern, Mallick Mohammad Aktaruzzaman

**Affiliations:** 1 Communicable Diseases Surveillance Unit, World Health Organization Country Office for Bangladesh, Dhaka, Bangladesh; 2 Improvement of Urban Public Health Preventive Services Project, Local Government Division, Ministry of Local Government, Rural Development and Co-operatives, Dhaka, Bangladesh; 3 Communicable Disease Control Unit, Directorate General of Health Services, Ministry of Health & Family Welfare, Dhaka, Bangladesh; 4 Institute of Public Health Nutrition (IPHN), Mohakhali, Dhaka, Bangladesh; 5 Communicable Disease Control Unit, Directorate General of Health Services, Ministry of Health & Family Welfare, Dhaka, Bangladesh; 6 Ghatail Upazila Health Complex, Ghatail, Tangail, Bangladesh; 7 Former Department of Medical Entomology, National Institute of Preventive and Social Medicine, Dhaka, Bangladesh; 8 Centre for Medicine and Society, University of Freiburg, Freiburg, Germany; 9 UNICEF/UNDP/World Bank/WHO Special Programme for Research and Training in Tropical Diseases, World Health Organization, Geneva, Switzerland; 10 Department of Communicable Diseases, World Health Organization Regional Office for South-East Asia, New Delhi, India; 11 Emerging Infections and Parasitology Laboratory, International Centre for Diarrhoeal Disease Research, Bangladesh (icddr, b), Dhaka, Bangladesh; 12 Department of Microbiology, Green-Life Medical College, Dhaka, Bangladesh; 13 Hospital Service Management Unit, Directorate General of Health Services, Ministry of Health & Family Welfare, Dhaka, Bangladesh; 14 Department of Medicine, Infectious Diseases Hospital, Dhaka, Bangladesh; 15 Infectious and Tropical Medicine Department, Mymensingh Medical College and Hospital (MMCH), Mymensingh, Bangladesh; 16 National Institute of Laboratory Medicine & Referral Centre, Dhaka, Bangladesh; 17 Dev Care Foundation, Dhaka, Bangladesh; 18 Directorate General of Health Services, Ministry of Health & Family Welfare, Dhaka, Bangladesh; 19 Leishmaniasis Programme, Drugs for Neglected Diseases initiative (DNDi), Geneva, Switzerland; 20 Control of Neglected Tropical Diseases, World Health Organization, Geneva, Switzerland; 21 Department of Epidemiology, University of California San Francisco, San Francisco, California, United States of America; FIOCRUZ Bahia: Instituto Goncalo Moniz, BRAZIL

## Abstract

In 2023, Bangladesh became the first country to achieve World Health Organization (WHO) validation of elimination of visceral leishmaniasis as a public health problem, defined as maintenance of annual kala-azar incidence at <1 case per 10,000 population at the subdistrict (upazila) level. The pillars of the programme are early diagnosis and effective treatment, indoor residual insecticide spraying, improved case detection, social mobilization and operational research, and effective disease surveillance. The Bangladesh National Kala-azar Elimination Programme was established in 2008, with introduction of rapid diagnostics and newer treatment modalities in health complexes at sub-district level in the endemic area in 2012–2015, initiation of blanket IRS in affected communities in 2012–2013 and adoption of a digital surveillance system in 2015. All subdistricts achieved and maintained the elimination threshold from 2017 onward. We present documentation of the course of KA elimination in Bangladesh and provide a perspective on the components necessary to maintain current success into the future.

## Author summary

In 2023, Bangladesh became the first country validated by the WHO for eliminating visceral leishmaniasis (kala-azar) as a public health problem—defined as having less than 1 case per 10,000 population annually at the subdistrict (upazila) level. The National Kala-azar Elimination Programme, launched in 2008, implemented key strategies: early diagnosis and treatment, indoor residual spraying (IRS), improved case detection, social mobilization, operational research, and digital surveillance. Rapid diagnostics and updated treatment protocols were introduced in subdistrict health complexes from 2012–2015, alongside blanket IRS campaigns and digital surveillance adoption in 2015. By 2017, all subdistricts had achieved and sustained the elimination threshold. Bangladesh’s success reflects strong programmatic coordination and evidence-based interventions. This document outlines the elimination journey and highlights essential components to sustain progress and prevent resurgence.

## Introduction

Visceral leishmaniasis (VL) is caused by protozoan parasites of the *Leishmania donovani* complex and transmitted by the bite of phlebotomine sand flies [1]. Kala-azar (KA) is the most severe manifestation of VL, characterized by prolonged fever, splenomegaly, hepatomegaly, and bone marrow invasion leading to pancytopenia and immunosuppression [1]. Without timely treatment, mortality occurs from secondary infections, severe anemia, hemorrhage, or simply advanced KA. VL was first described in 1824 in Jessore, now a district in Bangladesh, in an epidemic that reportedly killed 75,000 people over a 3-year period [2]. Fittingly, 199 years later, Bangladesh became the first country to achieve World Health Organization (WHO) validation of elimination of VL as a public health problem [3].

Until 2000, specific treatment for VL relied on the highly cardiotoxic pentavalent antimony preparations, that required daily injections for 28 days. [4]. Diagnosis relied on bone marrow or splenic aspiration, or the aldehyde test, a non-specific measure of hypergammaglobulinemia which appeared only after months of illness [5]. In early 2000s, sensitive point-of-care rapid tests and new treatment modalities, such as oral miltefosine and single dose liposomal amphotericin B (LAmB), which ensured better patient retention, were evaluated and proposed as modalities for enhanced control of the disease [6–9].

Historically, the global burden of human VL disease burden has been highest in the Indian subcontinent (ISC), East Africa and Brazil [10]. In the ISC and East Africa, VL is caused by *Leishmania donovani*, with transmissions considered entirely anthropometric [13]. Elsewhere, including the Mediterranean basin, western and central Asia, and Latin America, *L. infantum* circulates in sylvatic and domestic zoonotic cycles [11]. Dogs and other mammals such as hares act as reservoirs. In most settings, canine VL incidence is commoner than human VL incidence [12].

In the ISC, the recognized infection reservoirs consist of humans with KA and those with post-kala-azar dermal leishmaniasis (PKDL), a pleiomorphic dermatosis affecting 5%–15% of clinically cured kala-azar patients in the 5 years after treatment [1]. Patients with PKDL are not systemically ill, and diagnosis and treatment are challenging [13]. The rK39 rapid test can remain positive for years after treated KA and thus has poor specificity for PKDL diagnosis. Demonstration of the parasite is technically difficult, especially in the pauci-parasitic macular form, which accounts for the majority of PKDL in the ISC [14]. Treatment requires a prolonged drug course, formerly 120 days of Sodium Stibogluconate (SSG), or more recently, 84 days of miltefosine. Both drug courses carry a substantial risk of serious side effects [4,15]. Studies reveal that Kala-azar and PKDL patients can infect sand flies in laboratory experiments and untreated PKDL patients may act as long-term reservoirs especially during periods of low VL incidence. [16,17].

In the early 2000s VL elimination was considered feasible in the ISC, due to lack of non-human reservoirs, availability of rapid tests, and oral drugs [9]. In 2005, Bangladesh, India, and Nepal committed to eliminate VL as a public health problem in a Memorandum of Understanding (MoU) that was facilitated by the WHO South-East Asia Regional Office (SEARO) [9]. The Regional Strategic framework for kala-azar elimination (2005–2015), defines elimination as a public health problem as “maintenance of annual KA incidence at <1 case per 10,000 population at the designated geographic level (upazila [subdistrict; the average surface area is approximately 300 square kilometers] for Bangladesh, block for India, district for Nepal) for at least 3 consecutive years” [1]. This framework emphasized on decreasing human reservoirs through early diagnosis and effective treatment near the patients’ homes and decrease vector populations through integrated vector management through indoor residual spraying (IRS). The proof of progress was documented through strong disease surveillance and operational research.

After signing the MoU, the government of Bangladesh initiated activities toward the elimination initiative, which gained momentum in 2008. This article presents the detailed national surveillance data documenting the course of VL elimination in the country and offers a perspective on sustaining success in the future.

## Methods

### Ethics statement

Ethical approval was considered unnecessary as the surveillance data presented in the article were collected as part of routine public health activities.

### Search strategy and selection criteria for published or unpublished documents

Country-specific information was gathered based on the search terms “visceral leishmaniasis or kala-azar” and “Bangladesh.” Regional information on diagnosis, antileishmanial treatment, surveillance, and vector control was compiled through PubMed searches for each of those topics plus “visceral leishmaniasis or kala-azar” and “Bangladesh or India or Nepal.” We narrowed our search to articles relevant to the implementation of activities for VL elimination in Bangladesh. Related articles cited in these publications were reviewed. Articles other than Bangladesh were excluded. In addition, we relied on two monographs provided by the elimination programme and/or WHO, which provide specific data on the activities and progress of the VL elimination programme not available in the conventional literature [18,19]. We consider these data to be essential documentation for future planning and public health responses. We also reviewed WHO guidelines and recommendations related to VL elimination in the ISC.

### National surveillance data

The surveillance data utilized in the current study were collected either passively (1994–2014) or by a combination of passive and active case detection (2015–2023) by the National Kala-azar Elimination Programme of the Directorate General of Health Services. Prior to 2015, surveillance was paper-based at the upazila level and aggregated by districts for central reporting. Since 2015, data have been managed on the DHIS2 platform.

## Evidence and activities performed targeting elimination and current status

### Pre-MoU landscape of VL control (<2005)

*L. donovani* was identified as the causative agent of VL in 1903. The vector and the transmission cycle were described, and treatment with pentavalent antimony was started in the 1920s [2]. Major epidemics occurred in the 1920s and 1940s in the ISC, including areas of present-day Bangladesh [20]. Widespread DDT spraying during the global malaria eradication campaign in the 1950s–1960s led to a sharp, though poorly documented, drop in VL incidence, and this was later cited as evidence that VL elimination is possible as a public health problem [21]. India experienced intense VL resurgence with epidemic peaks in the late 1970s, early 1990s, and mid-2000s [22,23]. Similar resurgence was reported in the 1970s-1980s in Bangladesh, but with no systematic surveillance data available from the period, the precise epidemiological patterns remain unclear [24]. In 1998, Bangladesh terminated IRS with DDT due to environmental concerns, and thus there was a pause in systemic vector control efforts until 2012.

**Fig 1:**
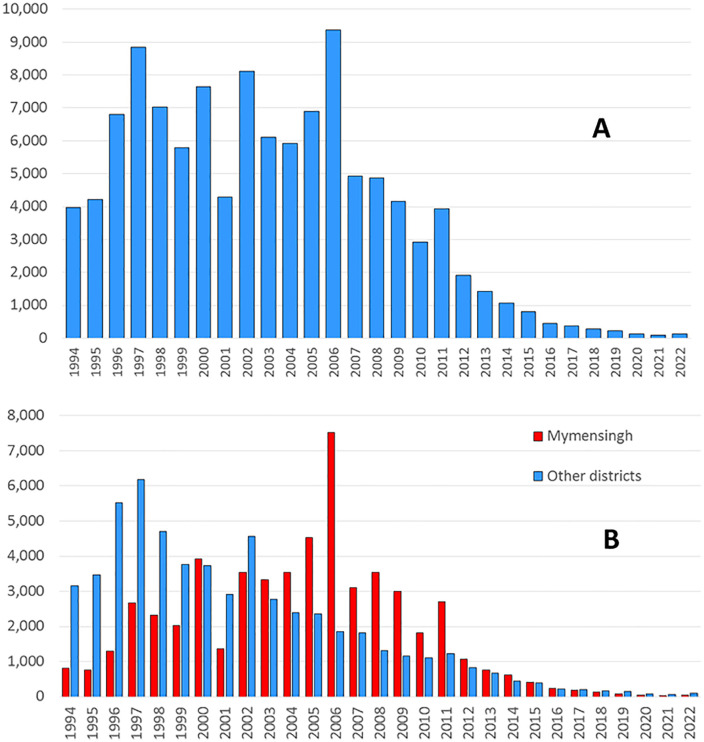
(A) Visceral leishmaniasis cases by year, Bangladesh 1994–2022. (B) Visceral leishmaniasis cases by year, Mymensingh district vs all other districts. All forms of kala-azar and PKDL in aggregate.

For Bangladesh, district-level VL surveillance data are available starting in 1994 (Fig 1A). These data come from the paper-based system which continued through 2014 [25,26]. VL cases were recorded at the time of diagnosis in hard copy registers in upazila health complexes (UHCs) and other health care facilities [27]. Aggregate numbers were then reported to the District Civil Surgeon offices, where they were further aggregated and transmitted to the national level. KA and PKDL cases were reported together in the case counts prior to 2014. National data from 1994 onward show a broad epidemic curve with peaks in 1997 and 2006. However, disaggregation by district tells a more nuanced story. The peak in the 1990s was due primarily to VL cases reported from Pabna, Tangail, Gazipur, Jamalpur and Sirajganj districts, whereas the peak in mid-2000s reflected an epidemic that disproportionately affected Mymensingh district (Fig 1B).

A research study conducted from 2002 to 2004 in a single village in Fulbaria, the most affected upazila at the time, provided community-level data on the pre-MoU situation, showing a cumulative incidence of 9%, with peak annual incidence of 200 VL cases per 10,000 in 2002 and 2003 [28,29]. At the time of the first survey, diagnosis in the UHC relied on the aldehyde test, which has high sensitivity only after 5–6 months of illness [5]. The rK39 rapid test was available only in private laboratories, where its price was equivalent to 10% of a village family’s average monthly income [30]. A major shortage of sodium stibogluconate (SSG) led to prolonged delays in treatment and drug prices up to 4 times the official figure [30]. From 1999 to 2001, the median symptom duration before treatment was 4 months. The KA case-fatality rate was 14% among female versus 5% among male patients [28]. By 2004, incidence was already falling in the study village but was said to be on the rise in neighboring communities (R. Chowdhury and C. Bern, personal observation, 2004). The occurrence of highly clustered local outbreaks, which burn out within a few years to be replaced by outbreaks in nearby communities, was subsequently described in India as well [31].

### Establishment of NKEP and research used to shape its strategy

The National Kala-azar Elimination Programme (NKEP) of DGHS was established in 2006 to operationalize the strategic objectives for achieving the elimination target. The government of Bangladesh was the primary source of funding, with additional support from other governmental and non-governmental sources, and technical and in-kind support from the World Health Organization. NKEP started by strengthening the capacities of service providers through training at primary and secondary level health facilities, i.e., doctors, nurses, laboratory technicians, etc., which was gradually scaled up to tertiary levels. NKEP also began efforts to ensure the required supplies, i.e., diagnostics, medicines, etc., at all levels. In the second half of the decade, studies in highly endemic districts highlighted the rise in PKDL cases [32,33]. The epidemic curve for VL peaked in 2006 followed by a peak in PKDL incidence two years later [14].

Operational research, conducted starting in 2006 with WHO TDR support, helped to spur the development of methodologies that were then incorporated into the elimination strategy. These projects focused on two major strategies: active case detection (ACD) and vector control. The yield of household level surveys, considered the gold standard for ACD, was shown to be strongly dependent on KA incidence [34]. Very high yield and low cost per case detected was demonstrated in Rajshahi district in 2006–2007, when incidence was at its peak and no ACD activities had been conducted. In this setting, house-to-house surveys detected one new KA case per 166 households screened. Contrasting this, the parallel survey in Muzaffarpur district in India detected one new case per 1432 households visited. The major difference between the two settings was the high accumulated case load in Rajshahi due to limited availability of diagnostic and treatment facilities prior to the study [34].

ACD strategies, including fever camps and case searches around known KA cases using the index case approach, were subsequently implemented and compared with household-level surveys. A multicountry study in India, Bangladesh, and Nepal found that the fever camp and index case approaches had aggregate sensitivities of 79% and 43%, respectively, though country-specific estimates showed wide variation with overlapping confidence intervals [35]. A cost benefit analysis study further revealed that fever camps was cost-effective in Bangladesh, with the cost per case detected of USD 22 [36]. In contrast, the cost were significantly higher in India and Nepal, due to the lower disease incidence in study sites, underscoring that as the KA incidence falls, the cost of ACD activities inevitably rises.

A comprehensive project was mounted by *Médecins sans Frontières*-Holland (MSF-H) covering Fulbaria upazila from 2010 to 2011. Activities included ongoing village-based ACD for KA and PKDL, plus diagnostic testing and treatment with LAmB (15 mg/kg in 3 divided doses for KA, 30 mg/kg in 6 divided doses for PKDL) [37]. The importance of ACD in finding untreated KA and especially PKDL patients was clear in the MSF data. Fulbaria (2010 population 442,244) and the neighboring upazila of Trishal (2010 population 403,845), where no such intervention occurred, reported VL incidence of 17.7 and 31.7 cases per 10,000 in 2009. During the 20-month project period, 1088 vs 756 KA cases, and 1145 vs 37 PKDL cases were treated in Fulbaria vs Trishal, respectively. Anecdotally, prior to the MSF-H project, many PKDL patients were reluctant to be treated by 120 days of intramuscular SSG (S. Islam, personal communication, 2008). It seems likely that patients were less reluctant to be treated by six IV infusions of LAmB, increasing self-referral as well as cases found through ACD in the MSF-H project.

A mathematical model developed during this time served to highlight the importance of shortening the time from onset of KA symptoms to diagnosis and effective treatment, in order to decrease the human reservoir of infection [38]. A later model, based on data from a cluster of highly endemic communities in Mymensingh, demonstrated that over the course of the decade from 2002 to 2012, KA was the dominant infection reservoir during the first half of the epidemic cycle but shifted to PKDL as KA incidence fell after 2006 [39]. Together with modeling based on data from India [40], these models support the central strategy of rapid KA diagnosis and prompt treatment with single dose LAmB as crucial in the achievement of elimination as public health problem and highlight the issue of PKDL detection and management as a key concern going forward.

Vector control was the second major focus of TDR-supported operational research from 2006 onward. The earliest multi-country cluster-randomized trial (CRT) was conducted in 2006–2007 compared indoor residual spraying (IRS), insecticide-treated nets (ITNs) and environmental management (EVM) to a control arm (no intervention) [41,42]. The data from Fulbaria, the Bangladesh study site showed that the sand fly density peaked in March, and the highest proportion of gravid females were in May [42]. The IRS and ITN arms were associated with a 70%–80% decrease in male and female *Ph. argentipes* density up to 5 months post intervention, whereas EVM showed no impact. Vector density rebounded by 11 months post-IRS, whereas ITN households continued to show significantly lower density compared with control households. The second CRT (2008–2009) examined feasibility, acceptability, and effectiveness of a bed net dipping program using slow-release insecticides (KO TAB 123) in Mymensingh and Rajshahi in Bangladesh [43]. The intervention achieved approximately 60% reduction in sandfly densities over 18 months and showed >80% sand fly mortality on bioassays. The third CRT (2011–2013) compared a commercial insecticide-treated durable wall lining (DWL), ITNs and indoor lime-containing wall wash [44]. Sand fly densities were significantly decreased in the ITN and DWL arms up to 9- and 12-months post-intervention, respectively. Vector mortality was higher and more sustained for DWL than ITNs. The wall wash had only a transient effect on both parameters. However, the cost of DWL was perceived to an important impediment to widespread deployment, running 3–4 times that of an ITN [45].

Recent studies have investigated innovative vector control approaches. An extensive CRT of multiple vector control modalities suggests that a combination of several methods may provide more benefit than any single modality [46]. The analysis was not powered to allow comparison between intervention arms, but outcomes better than any individual intervention were seen in three combination arms: IRS plus long-lasting insecticide-treated nets (LLINs), IRS plus KOTAB-impregnated nets, and LLINs plus the larvicide chlorpyrifos 20EC. IRS is much more costly in materials, human resources and logistics, suggesting that the LLIN-larvicide combination should be further evaluated for potential use in the post-elimination phase. Other novel interventions, such as insecticide-impregnated wall paint, should also be evaluated for impact and cost-effectiveness [47].

The data produced by the research outlined above directly informed the development of the NKEP elimination policy, which culminated in national guidelines, standard operating procedures and directives to support VL elimination efforts. Furthermore, NKEP implemented interventions based on the above-noted evidence, including ACD, roll-out of rK39 rapid tests, introduction of miltefosine and continued capacity strengthening in all endemic upazilas.

### Full implementation under the National Kala-azar Elimination Programme (2012–2022)

**Fig 2:**
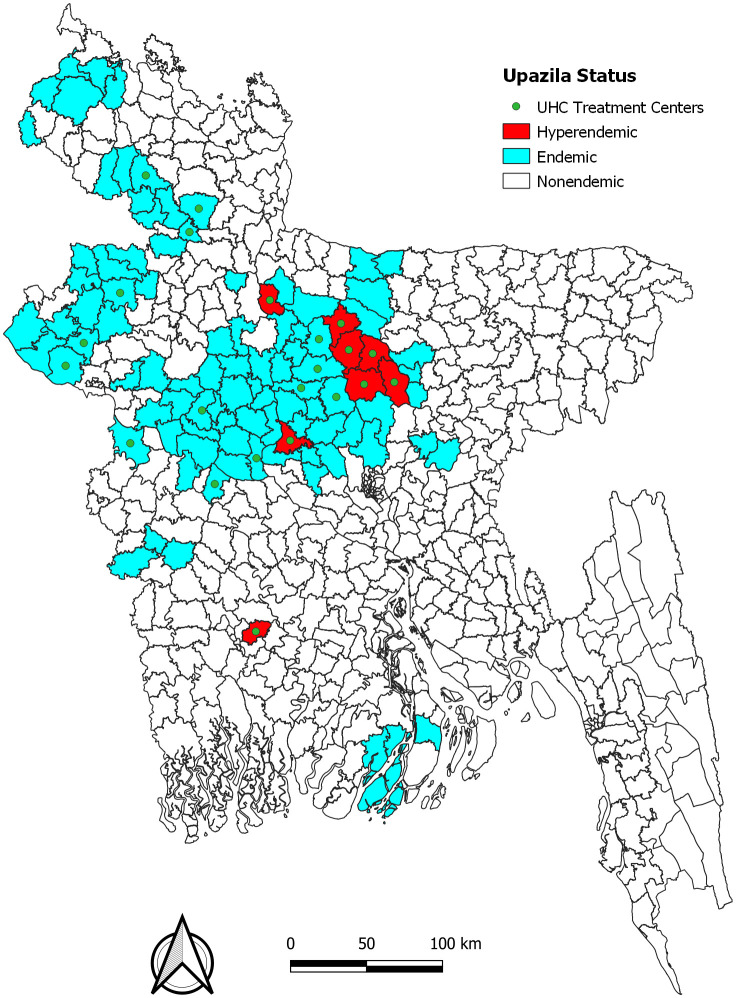
Map of upazila endemicity. High endemic upazilas in red, endemic upazilas in blue. Upazila health centers (UHCs, in green) provide primary diagnosis and treatment, and referral hospitals (red crosses) provide higher-level care. The maps were created for data visualization using QGIS (version 3.14.16) and the shapefile available at: https://data.humdata.org/dataset/cod-ab-bgd.

Between 1994 and 2011, VL cases were reported in 140 upazilas across 37 districts [48]. However, 16 upazilas in nine districts were responsible for the vast majority of reported cases. In 2011, NKEP selected 100 upazilas in 26 districts in which to target national strategic actions for achieving the elimination goal (Fig 2). The upazilas (sub-districts) where active transmission occurred were classified as endemic, totaling 100 in number. Those that consistently reported incidence rates above 2 per 10,000 population between 2008 and 2011 were classified as hyperendemic. Accordingly, eight upazilas were designated as hyperendemic (Fulbaria, Trishal, Gafargaon, Bhaluka, and Muktagaccha in Mymensingh district; Nagorpur in Tangail, Madarganj in Jamalpur, and Terokhada in Khulna). The top two upazilas (Fulbaria and Trishal) accounted for 52% of the cumulative national case load from 2008 to 2011. The eight hyperendemic upazilas were addressed earlier in the programme and tended to receive more intensive interventions than other upazilas.

**Fig 3:**
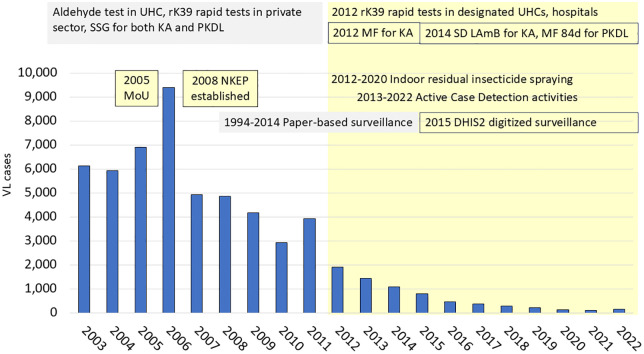
Timeline of activities and interventions since 2003. Period of systematic implementation of interventions under NKEP highlighted in yellow; pre-NKEP routine activities in gray. Abbreviations: NKEP, National Kala-azar Elimination Programme; UHC, Upazila Health Complex; KA, kala-azar; PKDL, post-kala-azar dermal leishmaniasis; MF, miltefosine; LAmB, liposomal amphotericin B.

Comprehensive implementation of most programme interventions was achieved for the first time in 2012–2014 (Fig 3). The marked decrease in incidence from 2006 to 2012, due in large part to the natural epidemic cycle [22], likely facilitated subsequent programme impact. Provision of rK39 rapid tests and intensive training of clinical personnel began with health care facilities in the 8 hyperendemic upazilas in 2012 and quickly expanded [18,19]. Drug treatment transitioned several times. Sodium stibogluconate (SSG) was the primary treatment for both KA and PKDL from the 1920s until the early 2000s throughout the region, when SSG resistance was highlighted in Indian publications [49]. SSG continued to be used in Bangladesh until 2008 when an attempt was made to transition to a 28-day course of miltefosine for KA based in part on RCT data generated in Mymensingh [50]. This transition was interrupted when frequent KA treatment failures were reported and the drug being used was found to contain no active ingredient [51]. SSG temporarily returned as the mainstay of KA treatment. As early as 2003, a single LAmB infusion was shown to yield cure rates >90% in clinical trials in India and Bangladesh, but use of this regimen was constrained by the high commercial cost of the drug [8,52–54]. In 2014, WHO negotiated a 5-year donation of LAmB from the manufacturer, Gilead [55]. Simultaneously, there were reports of declining effectiveness of miltefosine for KA in the Indian subcontinent [56]. In 2014, NKEP transitioned to LAmB 10 mg/kg in a single 4-hour infusion as first line treatment for KA, based on efficacy of >97% in a Phase IV study in rural Mymensingh district [54]. The first-line treatment for PKDL was updated to 84 days of miltefosine [57]. First line drugs and personnel trained to use them were made available in 22 upazila health complexes plus 7 referral centers [19].

Deltamethrin IRS was instituted in 2012 in the 8 hyperendemic upazilas using centrally-provided microplans based on the village-level number of reported cases [48]. In 2014, IRS expanded to all endemic upazilas [18]. Although IRS was conducted every year from 2012 to 2020, not all upazilas were included each year, and in some years, only one of the two prescribed rounds was conducted. IRS was not performed from pre-monsoon 2020 till pre-monsoon 2023 due to the unavailability of insecticide. In 2012 and 2013, NKEP also provided a total of 50,000 LLINs to KA and PKDL patients treated in government health care facilities in or after 2008 [19]. Vector surveillance was patchy and heterogeneous, making the entomological impact of interventions impossible to assess directly [19].

ACD based on the camp and index case approaches was instituted in 2012 [18] and even more rigorous camp-based ACD continued till 2023. House-to-house searches were also undertaken in 2013 and 2014. The 2017 WHO Joint Monitoring Mission (JMM) committee raised questions about the sensitivity of these ACD exercises and lack of systematic follow-up of identified VL suspects. On the other hand, community awareness of VL and access to diagnosis and treatment were noted to be high [18,58]. As NKEP repeatedly conducted community sensitization activities in the endemic communities using several tools, i.e., leaflet, poster, billboard, miking, docudrama, uthan baitak (small group discussion in the village), folk song, etc. NKEP transitioned from paper-based surveillance to the fully digital DHIS2 system in 2015, after piloting its feasibility testing and operationalization by providing training to concerned officials at UHCs and CS Office at districts [personal communication, D Mondal], facilitating the evaluation of progress toward the elimination target [19].

### Achievement and maintenance of the public health elimination of VL since 2017

**Fig 4:**
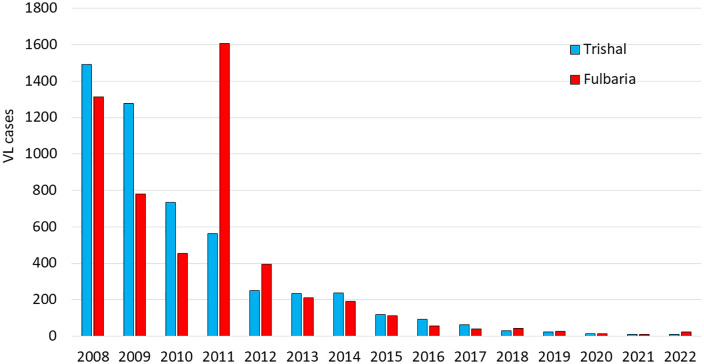
Visceral leishmaniasis cases by year in Fulbaria and Trishal Upazilas, Mymensingh district. All forms of kala-azar and PKDL in aggregate. The MSF-H project ran from 2010 to 2011.

**Fig 5:**
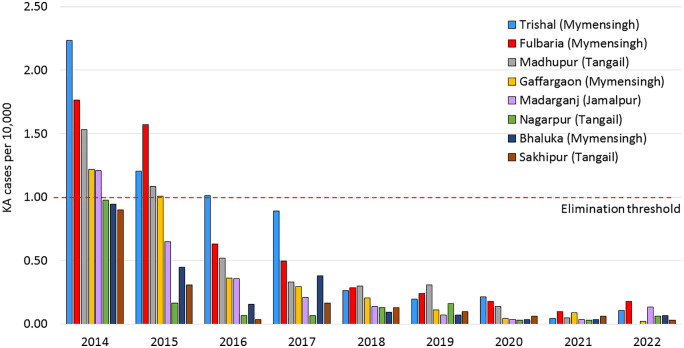
Annual kala-azar (KA) incidence in the 8 most affected upazilas. Incidence calculated as new KA cases plus KA relapse cases divided by upazila population projected for corresponding year, expressed as KA cases per 10,000.

Reported case numbers declined every year from 2008 to 2022, except for an increase from 2010 to 2011. This temporary increase in aggregate case numbers may have resulted in part from the MSF-H project in Fulbaria (Fig 4) [37]. By 2017, incidence in all upazilas was below the elimination threshold and has remained so ever since (Fig 5). VL caseloads have been very low since 2018, with a total of only 879 VL cases (including all forms of KA plus PKDL) during the 5-year period of 2018–2022 compared to 15,878 in the 4 years from 2008 to 2011, corresponding to >94% decrease in disease burden [19]. Given the systematic surveillance implemented under DHIS2 and recent ACD activities, the caseload for 2008–2011 likely represents a substantial underestimate compared to much greater completeness for 2018–2022. Table 1 shows the decline in the number of reported new KA cases, indicating reduced transmission. Consequently, in October 2023, Bangladesh became the first country in the world to achieve the historic milestone of validating the elimination of visceral leishmaniasis as a public health problem [3].

**Table 1:** Surveillance data disaggregated by form from 2014 to 2024, Bangladesh.

Year	Total	New KA	Relapse KA	KA Treatment Failure	PKDL
2014	1068	654	85	11	318
2015	796	493	67	3	233
2016	454	259	39	2	154
2017	376	210	61	3	102
2018	289	124	52	1	112
2019	223	97	30	3	93
2020	129	46	25	0	58
2021	98	35	23	3	37
2022	142	47	17	1	77
2023	142	34	22	1	85
2024	87	23	19	0	45

## Remaining questions and evidence gaps

The KA elimination programme in Bangladesh has been a striking success. Nevertheless, fundamental questions remain concerning transmission dynamics of *L. donovani*, vector bionomics, the impact of the various interventions, and whether elimination of transmission is possible with current tools [22,59]. Locations of higher VL incidence have remained fairly consistent over the time for which data are available: 8 of the 10 upazilas with the highest caseload in 2008–2011 remained among the top 10 most affected upazilas in 2018–2022 data. However, the top 10 upazilas accounted for 77% of all VL cases from 2008 to 2011 compared to 52% for the later time period, and single isolated KA cases were reported in both endemic and non-endemic upazilas [19]. This pattern raises the question of how anthroponotic VL transmission can be maintained under such low incidence conditions. Potential cryptic infection sources include undetected KA and/or PKDL patients, low-level transmission from asymptomatically infected individuals and the possibility that one or more non-human animal species might act as secondary reservoir hosts. Resolution of these questions is crucial to sustaining the current achievement of excellent VL disease control as well as the aspirational goal of eliminating VL transmission in the future.

Molecular data show a major genetic bottleneck for South Asian *L. donovani*, corresponding to the 1950s-1960s when blanket DDT spraying aimed at malaria eradication is thought to have led to quasi-elimination of transmission [60]. During the subsequent VL resurgence, investigators postulated that PKDL patients constituted the most important inter-epidemic reservoir, carrying over a subpopulation of parasites from the epidemic cycle of the 1940s into the 1970s [61]. The regional programme never instituted an elimination target for PKDL, which remains a challenge for passive detection, sensitive diagnosis and effective treatment [13]. In 2020–2022 surveillance data, PKDL cases outnumber primary KA cases. If ACD activities are no longer supported, PKDL detection will decline and many untreated PKDL patients may remain in communities as potential sources of resurgence.

Two other possible hidden reservoirs include infected non-human animals, especially dogs, and humans with asymptomatic leishmanial infection. Current data are insufficient to confirm or refute their roles in the VL transmission cycle in the ISC. Dogs are the main infection reservoir for *L. infantum* in Europe and the Americas [62,63], but whether they play a role in *L. donovani* transmission is unknown. The only published canine data from Bangladesh are small studies with surrogate indicators [64,65]. However, data from xenodiagnosis of symptomatic, PCR-positive dogs in India [66] raise the possibility that canine VL could act as a potential infection reservoir in the ISC, as they may do in the East African *L. donovani* transmission cycle [67,68]. Substantially more data will be needed to clarify their importance in parasite transmission. In a xenodiagnosis study in India, none of 184 asymptomatic humans with serological evidence of infection were infective to sand flies [17,69]. However, the sensitivity of xenodiagnosis is less than perfect, and the sample size was not large enough to rule out transmission from a small percentage of such infections.

No controlled trial of IRS has ever been conducted in the ISC, making its effectiveness as a public health intervention impossible to define, and the fact that incidence had already fallen by 80% (from 9379 in 2007 to 1902 in 2012) before the initiation of IRS raises questions about its contribution to VL elimination. The question of effectiveness is now unanswerable, since current very low incidence would necessitate an unfeasibly large sample size. In any case, the high resource requirements of large-scale IRS necessitate a new approach to vector control in the post-elimination era. In 2010, the KalaNet trial, the only cluster-randomized ITN trial in the ISC with a clinical outcome, failed to show an impact on either leishmanial infection or KA incidence [70]. This trial was conducted in India and Nepal; the results led both countries’ national programmes to conclude that ITNs were ineffective and exclude them from their vector control strategy. By contrast, two *post hoc* analyses of Bangladesh KA surveillance data in sites of earlier net distribution demonstrated a significant decrease in KA incidence [71,72]. There are obvious methodological issues, but also biological and behavioral issues that may lead to differences in the impact of ITNs in different locales. In India, several recent studies have shown that *Ph. argentipes* may be substantially more exophilic and exophagic than previously assumed, behavior that may have been influenced by the decades during which patchy IRS with DDT continued to be conducted despite increasing evidence for vector resistance [73,74]. Comparisons of indoor vector density in sprayed vs unsprayed villages also failed to show any impact of IRS in India [75]. Furthermore, recent epidemiological analyses suggest that sleeping outside and early morning outdoor exposure during open defecation may increase risk of KA in India [76]. In Bangladesh, the complete suspension of IRS from 2005 to 2012 may have inadvertently maintained insecticide susceptibility, as well as the indoor feeding and resting habits of the vector, promoting effectiveness of pyrethroid IRS when it was instituted after 2012. Answering these questions would require a well-designed survey comparing the bionomics of *Ph. argentipes* in Bangladesh vs India; systematic harmonized vector surveillance would be even more useful but challenging to mount and maintain.

## Conclusion

NKEP recently finalized the National Strategic Plan for Kala-azar Elimination in Bangladesh 2020–2030: Towards Kala-azar Free Bangladesh (NKEP-DGHS 2022). Under this plan, the government of Bangladesh has committed to post-validation activities to guard against resurgence of KA and work toward elimination of VL transmission. In the absence of a proven vaccine, strong surveillance for case clusters and PKDL, and maintenance of preventive measures will be crucial (Box 1). Planning now, during the nadir of VL incidence, is important to enable prompt detection of impending VL resurgence, and to avoid excess morbidity and mortality in the future.

**Box 1. Lessons for the post-elimination future**

- **Sustain effective level of VL and PKDL surveillance**
  - Maintenance will be facilitated by transition to an integrated surveillance approach, including syndromic surveillance for febrile illness and skin conditions
  - Enhancements (e.g., data on time from symptom onset to treatment) should be considered
  - Include systematic outbreak investigations, investigation of cases in non-endemic upazilas and cross-border surveillance
  - Incorporate automated flags into the system (e.g., trigger to suggest field investigation if >2 KA and/or PKDL cases are reported in a union within 1 year)
- **Maintain VL case management expertise and material resources for clinical needs**
  - Ongoing training modules incorporated into routine continuing medical and public health education to ensure clinical recognition and ability to respond to VL cases
  - Maintain diagnostic and treatment supplies in stock in strategically located facilities
  - Ensure sufficient funding at the Ministry level
- **Transition to sustainable vector control and vector surveillance strategies**
  - Control based on integrated vector management
  - Establish sentinel vector surveillance system with capability to respond flexibly to changing circumstances (e.g., case clusters)
  - Critical evaluation of the role of IRS in the post-elimination setting, and introduction of other vector control tools
- **Document programme activities to enable effective evaluations in the future**
  - Document future VL control activities thoroughly when they occur
  - Maintain resulting data in compatible digital formats that can be combined and accessed to provide a full picture over time
  - Design vector control and clinical interventions to enable assessment of impact on disease incidence, separately and in combination
- **Research to elucidate crucial unanswered questions should be funded and conducted sooner rather than later, for example**
  - Research to develop innovative methods to maintain vector control and appropriate case management
  - Preparation to be able to include in-depth investigation of extent of transmission and infection sources as part of case cluster response
  - Assessment of the feasibility of elimination of transmission and a carefully designed demonstration project to identify the best combination of interventions

**Five key papers**

- Bern C, Chowdhury R. The epidemiology of visceral leishmaniasis in Bangladesh: prospects for improved control. The Indian journal of medical research. 2006;123(3):275–88. PubMed PMID: 16778310.
- Bern C, Hightower AW, Chowdhury R, Ali M, Amann J, Wagatsuma Y, et al. Risk factors for kala-azar in Bangladesh. Emerg Infect Dis. 2005;11(5):655–62. Epub 2005/05/14. https://doi.org/10.3201/eid1105.040718. PubMed PMID: 15890115; PubMed Central PMCID: PMC3320384.
- Joshi AB, Das ML, Akhter S, Chowdhury R, Mondal D, Kumar V, et al. Chemical and environmental vector control as a contribution to the elimination of visceral leishmaniasis on the Indian subcontinent: cluster randomized controlled trials in Bangladesh, India and Nepal. BMC medicine. 2009;7:54. Epub 2009/10/07. https://doi.org/10.1186/1741-7015-7-54. PubMed PMID: 19804620; PubMed Central PMCID: PMCPMC2763005.
- Chowdhury R, Mondal D, Chowdhury V, Faria S, Alvar J, Nabi SG, et al. How far are we from visceral leishmaniasis elimination in Bangladesh? An assessment of epidemiological surveillance data. PLoS Negl Trop Dis. 2014;8(8):e3020. Epub 2014/08/22. https://doi.org/10.1371/journal.pntd.0003020. PubMed PMID: 25144317; PubMed Central PMCID: PMC4140646.
- Chowdhury R, Faria S, Huda MM, Chowdhury V, Maheswary NP, Mondal D, et al. Control of Phlebotomus argentipes (Diptera: Psychodidae) sand fly in Bangladesh: A cluster randomized controlled trial. PLoS Negl Trop Dis. 2017;11(9):e0005890. Epub 2017/09/06. https://doi.org/10.1371/journal.pntd.0005890. PubMed PMID: 28873425; PubMed Central PMCID: PMCPMC5600390.
